# Yang-Monti Continent Ileovesicostomy: Experience with Three Cases

**Published:** 2011-07-30

**Authors:** Yogesh K. Sarin

**Affiliations:** Department of Paediatric Surgery, Maulana Azad Medical College New Delhi, India.

**Keywords:** Mitrofanoff, Appendicovesicostomy, Ileovesicostomy

## Abstract

Mitrofanoff appendicovesicostomy has been the method of choice for dealing with urinary incontinence. However, there may be some cases where some alternate conduits have to be used. Yang-Monti ileovesicostomy is an alternative to Mitrofanoff appendicovesicostomy. Three boys who underwent successful Yang-Monti continent ileovesicostomy are reported in this manuscript. In the first case, Mitrofanoff procedure was done for traumatic anorectal and urethral disruption after attempting ureterosigmoidostomy. Later on, on the request of the patient the appendicovesicostomy was excised. The patient presented later with chronic renal failure and bilateral hydroureteronephrosis thus an ileovesicostomy was fashioned. The patient could not be survived due to chronic renal failure related complications. In the second patient with exstrophy of bladder, the ileocecal junction along with appendix had to be resected on account of strangulated inguinal hernia; later on, an ileovesicostomy was performed for small capacity bladder and major degree of vesicoureteric reflux. The third patient with exstrophy of bladder, in whom Mitrofanoff procedure had been performed, presented with stenosis of the appendicovesicostomy. On re-operation the entire channel had disappeared thus necessitated ileovesicostomy. Both of these patients were doing well on follow-up.

## INTRODUCTION

Ever since the Mitrofanoff principle of continent catheterizable channel was introduced in 1980, surgical reconstruction for urinary incontinence has been revolutionized. Appendicovesicostomy has become the conduit of choice for Mitrofanoff procedure in the last 3 decades. However, there are several circumstances where an alternative is required. We describe such situations in three of our patients where Yang-Monti continent ileovesicostomy was successfully done. 

## CASE REPORT

**Case 1: ** A 10-year-old boy sustained an accidental gunshot injury resulting in a shattered perineum. He had pelvic fracture with anorectal as well as urethral disruption. He underwent an emergency left transverse colostomy and a suprapubic cystostomy. Later, in another hospital, the ureters were re-implanted into the sigmoid colon that was used as an incontinent urinary reservoir. The upper end of descending colon (distal to the transverse colostomy) was obliterated with a non-absorbable ligature.

The child presented to us for urinary undiversion. Through Posterior sagittal approach anorectoplasty and perineal reconstruction was done. Urethral repair was also performed by end-to-end anastomosis. The ureters were re-implanted into the defunctionalised urinary bladder (Cohen’s method) and a Mitrofanoff procedure using appendiceal conduit was performed. Subsequently the colostomy was closed. The patient refrained from doing clean intermittent catheterization (CIC) through either conduits (urethra and appendicovesicostomy) in the sheer delight of being once again able to pass urine per urethra after 1 year of initial injury. Six months later, the Mitrofanoff appendicovesicostomy was excised on request. The child was lost to follow up. 

Five years later, he presented with hypertension, chronic renal failure and right-sided optic nerve atrophy. There was bilateral hydroureteronephrosis on abdominal ultrasound. He was diagnosed to have neurogenic bladder (hypocontractile) with a large post-void residual urine volume. Voiding cystourethrogram revealed major degree vesicoureteric reflux on both sides. Cystourethroscopy demonstrated the urethra to be normal. He was advised CIC per urethra but was not accepted as it produced pain, being sensitive’ urethra.

Bilateral ureteral re-implantation was done. Since the appendix used for Mitrofanoff appendicovesicostomy had already been excised, a continent catheterizable stoma was fashioned using the Yang-Monti principle. A 2 cm segment of ileum was mobilized with a well vascularized mesentery. The ileal segment was divided longitudinally on its antimesenteric border. The opened bowel was then tubularized over a 12 F catheter along the long transverse axis, perpendicular to the mesentery. This was done in two layers, using fine absorbable sutures for mucosal approximation followed by a second serosal layer. The ends were closed with interrupted sutures while the middle part was closed with a running suture. The end result was a lengthened segment of bowel, about 7 cm long, with a perpendicular vascular pedicle (Fig. 1). This tube was re-implanted into the bladder as in appendicovesicostomy. 

**Figure:**
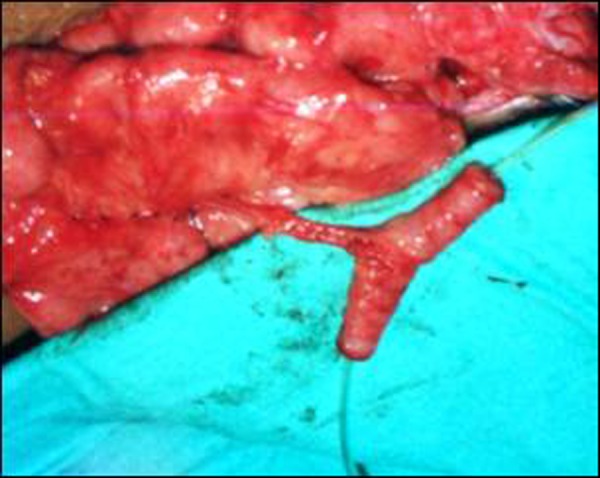
Figure 1: Intra-operative photograph showing ileal graft appearing very similar to vascularized appendicular graft generally used for MACE or Mitrofanoff procedures.

The patient was advised CIC through Yang-Monti channel while awake and continuous bladder drainage at night. Though he religiously followed this advice, his compliance to other medical treatment was low. He was being treated for long time by religious leaders and quacks. This worsened his renal functions drastically. At the age of 19 years, he was advised renal transplantation, but there were no willing donors. For next two years, he required biweekly hemodialysis. At age 21, he died of chronic renal failure and its related complications.

**Case 2:** A boy with exstrophy bladder and double phallus was seen at birth. The phallus in continuity with the urinary bladder was found to be rudimentary. At the age of three months, he developed strangulation of right inguinal hernia, which led to resection of gangrenous ileo-cecal junction along with the appendix. He underwent primary bladder closure after six months. 

At the age of four years, he was diagnosed to have major degree of vesicoureteric reflux on the left side with a small capacity bladder. As the appendix was lost at previous surgery, Mitrofanoff appendicovesicostomy could not be performed. Hence, Yang-Monti ileovesicostomy, using the same technique as described in case 1 was performed. At a later stage, genitoplasty was done. He was also advised daytime CIC and continuous bladder drainage at night. He has been doing well on follow-up as regards the renal functions and the social acceptability and the Yang-Monti channel has been complication-free since the last one decade.

**Case 3:** An eight-year old boy presented with repaired exstrophy bladder, done elsewhere, at the age of one year. He had been leaking urine through the wide penopubic fistula all these years. No attempt had been made to reconstruct his epispadiac penis. On investigations, he was diagnosed to have small capacity bladder (approximately 20cc) and preserved renal tracts. Bladder augmentation with colon, Young-Dees-Leadbetter bladder neck reconstruction along with Mitrofanoff procedure was done. Due to the small size of the native bladder and unusual configuration of the vermiform appendix, the latter was implanted in the bowel ‘augment’ and not the native bladder. The caecal end of the Mitrofanoff conduit was implanted in the ‘augment’ and the appendicular tip was brought out at the skin surface. The postoperative course was uneventful.

The patient returned after 8 months with stenosed Mitrofanoff channel. He had again started leaking from the penopubic fistula suggesting a failed bladder neck reconstruction. He was readmitted and prepared for the revision of Mitrofanoff channel, reconstruction/ closure of bladder neck and epispadias repair. At exploration, the capacity of the augmented bladder was satisfactory, but surprisingly the entire Mitrofanoff channel had disappeared. A continent catheterizable stoma with ileum was fashioned using the Yang-Monti principle. One important intra-operative complication worth mentioning here is that vascular pedicle of initial Yang-Monti channel was accidentally damaged by an assistant during surgery. A second Yang-Monti channel was similarly constructed with much ease. Bladder neck closure and Ransley’s repair of epispadias was done.

The child has been followed for 2 ½ years since the last surgery. He has been doing well on daytime 3 hourly CIC, daily bladder wash and night time bladder drainage through Yang-Monti channel.

## DISCUSSION

The main advantages of the appendix are a good blood supply, satisfactory lumen, stoma shape and auto-lubrication (it produces mucus). However, there are several circumstances where an alternative is required; either when the appendix is congenitally absent or unsuitable for use (short, kinked, partly stenosed, limited mobility of mesentery). It may have been removed or used previously (as was the situation in our two cases). Moreover, many children undergoing continent reconstruction of their urinary tract also suffer from fecal incontinence or chronic constipation.

A Malone antegrade continent enema (MACE) can be performed at the same time as urinary tract procedure. The appendix is far superior to other options in creating the MACE channel, and should probably be reserved for this purpose in patients with combined bladder and bowel incontinence. On rare occasions the appendix may be of adequate length to allow splitting into 2 segments, which are used to fashion a MACE channel and a Mitrofanoff conduit simultaneously [1]. The other disadvantage of using appendix for Mitrofanoff conduit is its small caliber requiring small catheters to empty the reconstructed bladder. As a result, poor bladder emptying and mucous pooling may occur, leading to increased incidence of bladder stone formation and spontaneous bladder perforation. Above all, the risk of inflammation due to appendicitis is always present and has been observed [2].

Though the stenosis of the Mitrofanoff channel is a known complication, its disappearance due to compromised blood supply as seen in our case 3 has never been reported. The unusual configuration of vermiform appendix and our choice of putting the appendix the other way around may have contributed. 

Other tubular structures used as alternatives to appendix are ureters, tapered ileum, tubularized flaps of urinary bladder, cecum and stomach; fallopian tube, vas deferens and preputial tube. In 1993, Yang described a technique of transverse retubularization of ileum to create a continent catheterisable conduit [4]. In 1997, Monti et al further studied this technique in a canine model. Since then, others have successfully performed transverse retubularized ileovesicostomy continent urinary diversion with good results. Advantages of Yang-Monti ileovesicostomy include constant availability, need for a very short segment of bowel, adequate mobility of the conduit and its mesentery, reliable vascularity, adequate channel length (multisegment channel) and diameter (better mucus clearance with larger catheters), and preservation of appendix for simultaneous or future MACE procedure. Moreover, if a bladder augmentation is also needed, the mesentery to the catheterizable stoma and the bowel for augmentation can be mobilized together. Modifications to increase the length of the channel when necessary, include transverse retubularized sigmoidovesicostomy, use of two ileal segments anastomosed at their mesenteric ends; and a technique dividing the bowel into two segments for 80% of its circumference, unfolding and reconfiguring as a single long channel (double in length) [5, 6]. Narayanswamy et al have reported a higher incidence of catheterization problems with the Yang-Monti conduit when compared to appendicovesicostomy [7]. However, others have reported lower incidences of postoperative complications requiring additional procedures, when the Monti conduit was fashioned [5, 6, 7].

Use of ureters has been shown to result in higher risk of complications due to associated ureteral reconstruction as well as a greater incidence of stomal stenosis [8]. A ‘continent vesicostomy’ has the advantage of avoiding intra-peritoneal surgery, large conduit diameter and preservation of appendix. However there is a significantly increased risk of stomal stenosis [4]. A gastric tube causes peristomal skin breakdown due to gastric secretions and is hence unacceptable [9]. Use of vas deferens and fallopian tube have been reported only anecdotally, have high revision rates (>40%), and should be reserved as salvage options [10]. Krstic has described the use of preputial tube successfully; however, this conduit is obviously unavailable in a female or a circumscribed male patient [11].

We conclude that the Yang-Monti channel can be considered an ideal alternative to the appendix. It is easy to create; producing a good caliber tube with minimal loss of bowel length, and it configures the valvulae connivantes in the longitudinal axis of the conduit, facilitating easier catheterization. As the conduit is on a mobile mesentery, there is no restriction on siting the abdominal wall stoma. 

## Footnotes

**Source of Support:** Nil

**Conflict of Interest:** None declared
